# The impact of Covid-19 on animal-assisted interventions: perceptions of UK animal-assisted intervention providers

**DOI:** 10.1093/pubmed/fdac126

**Published:** 2022-11-20

**Authors:** Emily Shoesmith, Selina Gibsone, Elena Ratschen

**Affiliations:** Department of Health Sciences, University of York, York YO10 5DD, UK; Dogs for Good, The Frances Hay Centre, Blacklocks Hill, Banbury OX17 2BS, UK; Department of Health Sciences, University of York, York YO10 5DD, UK

**Keywords:** COVID-19, health promotion, lockdown

## Abstract

**Background:**

Animal-assisted interventions (AAIs) are increasingly common in UK health settings. The Covid-19 pandemic has impacted on their delivery, with many organizations offering AAIs virtually during lockdown periods. This small-scale survey aims to explore the impact of Covid-19 on the delivery of AAIs, and associated challenges and opportunities.

**Methods:**

A cross-sectional, retrospective questionnaire survey was conducted with UK AAI providers. The anonymized survey was distributed via academic and third sector networks and social media. Descriptive statistics and free-text responses are presented.

**Results:**

Thirty-six AAI providers completed the survey. Of these, 83.3% continued to deliver AAIs during the pandemic. Twenty-eight delivered AAIs remotely and highlighted associated challenges, such as clients being unable to touch the animal, and clients having restricted access to the required technology. Over half reported their animal missed face-to-face interaction. However, they also reported advantages to remote delivery, such as for those who are allergic or fearful of animals. The most commonly reported challenges of *in situ* delivery included difficulty maintaining distance from the client and the use of face masks, which were perceived to hinder communication.

**Conclusion:**

The transition to remote delivery has highlighted challenges and opportunities. Further research could explore these in greater depth and compare the impact of different delivery modes.

## Introduction

The Covid-19 pandemic has caused a widespread shift in health services delivery from in-person to virtual platforms.^1–3^ This has also affected the delivery of animal-assisted interventions (AAIs), defined as interventions that involve an animal in structured or unstructured activities aimed at improving patients’ well-being.^3–5^ Virtual delivery constitutes a substantial deviation from the way AAIs are usually provided, based on an inherent sense that ‘live interaction’ including sensory engagement with the animal (e.g. through touch) are essential intervention components.^6–10^

Virtual AAIs may be reasonable to implement when *in situ* AAIs cannot be delivered,^11^ and may have similar benefits to traditionally provided AAIs. For example, viewing videos of animals and interacting with virtual animals have been found to have a positive impact on well-being and anxiety.^12–15^ Due to their convenience and cost-effectiveness, research has suggested virtual platforms should continue to be used.^16^ However, there is still a lack of understanding in relation to virtual AAIs compared with *in situ* delivery.^17^ It is important to further explore the impact of Covid-19 on AAI delivery, and in particular, explore the practicalities of transitioning to remote delivery. To the best of our knowledge, no survey studies have been conducted in this area during Covid-19. Results from this small-scale survey will enhance our preliminary understanding of the limitations of remote delivery, and AAI providers’ opinions on the associated challenges.

## Methods

### Study design

A cross-sectional, retrospective survey was conducted among UK AAI providers.

### Measures

A questionnaire was developed by academics with input from a UK assistance/therapy dog organization. The survey focused on the impact of Covid-19 on AAI delivery and the associated challenges, as detailed below.

Demographic data: Demographic information was collected about participants’ age (in bands), gender (male, female, non-binary) and current role (professional animal handler, volunteer, occupational therapist, physician, social worker, other).

Pre-pandemic AAI delivery: Participants were asked what kind of AAI they were providing before the pandemic (e.g. activities, therapy, education), and which animals were involved (e.g. dogs, cats, small mammals, horses, reptiles). Participants were asked to indicate whether they offered these interventions to groups or individuals, and how frequently they were delivering these interventions.

AAI delivery during the pandemic: Participants were asked if they were still providing AAIs since the Covid-19 outbreak. For those still delivering AAIs, participants were asked to indicate what type of interventions they were offering (remote only, *in situ* or both), and how frequently these were delivered. Participants were then asked to indicate their agreement with several statements relating to restrictions they experienced when delivering interventions *in situ* and/or remotely. For those delivering remote AAIs, statements included ‘Clients miss being able to touch the animal’ and ‘Clients have had restricted or no access to required technology’. For those delivering AAIs *in situ*, statements included ‘maintaining distance from the animal has been difficult’ and ‘clients are unable to touch or be close to the animal’.

Participants were also asked if the restrictions and provision of interventions had a perceived impact on their animal and if so, to indicate how their animal had been impacted among five pre-specified options, or to specify it themselves. The pre-specified options included: ‘there has been no change’; ‘my animal is missing face-to-face interaction’; ‘my animal is unsettled by the added safety measures’; ‘my animal is not unsettled by the added safety measures’ and ‘my animal’s attitude/behaviour has changed due to the change in work routine’. A question on whether participants had felt they were more or less satisfied with the provision of interventions since the change in practice was included.

There was also an open-ended question for comment on personal perspectives: ‘please share any further comments on your personal perspectives regarding the provision of animal-assisted interventions during the Covid-19 pandemic’.

### Recruitment and procedures

The anonymized survey was released in Qualtrics survey software and promoted using academic and third sector networks and social media. Prospective participants followed a link to the survey where they were presented with a Participant Information Sheet and consent form. Consent to participate in the survey was indicated by ticking an online check box. All data were stored on the Qualtrics sever at the University of York.

The study commenced in March 2021, during the third UK lockdown phase, and ended in May 2021, when lockdown measures were officially eased. Ethical approval for the survey was granted by the Health Sciences Research Ethics Committee at the University of York, UK, on 17th March 2021. Descriptive statistics and thematic analysis of free-text responses are presented.

## Results

Thirty-six participants completed the survey. Twenty-nine (80.6%) were female and the age ranged from 25 to 65 years. Sixteen (44.4%) were volunteers, followed by 13 professional animal handlers (36.1%). Various AAIs were delivered pre-pandemic, including therapy (61.1%; *n* = 22), activities (e.g. care home visits) (83.3%; *n* = 30) and education (e.g. reading programmes in schools) (36.1%; *n* = 13). Almost all providers worked with dogs only (80.6%; *n* = 29) and delivered their sessions to groups on a weekly basis. Four providers (11.1%) worked with a combination of dogs, cats, small mammals and reptiles, two (5.6%) worked with dogs and small mammals and one provider worked with dogs and cats.

### Impact of Covid-19 on AAIs

**Fig. 1:**
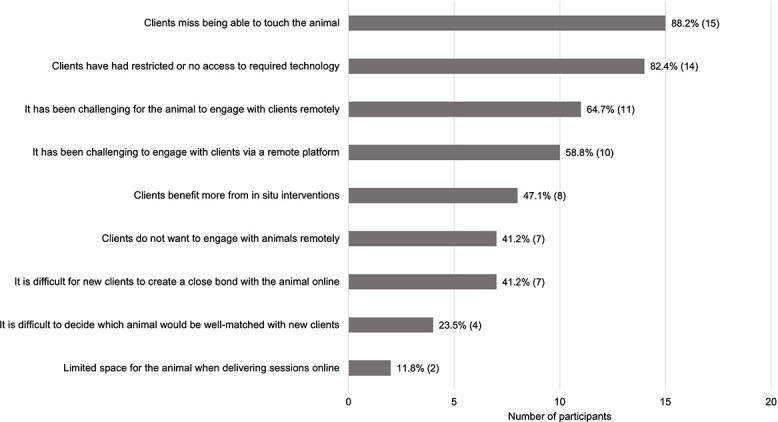
Providers perceptions of challenges when providing AAIs remotely.

**Fig. 2:**
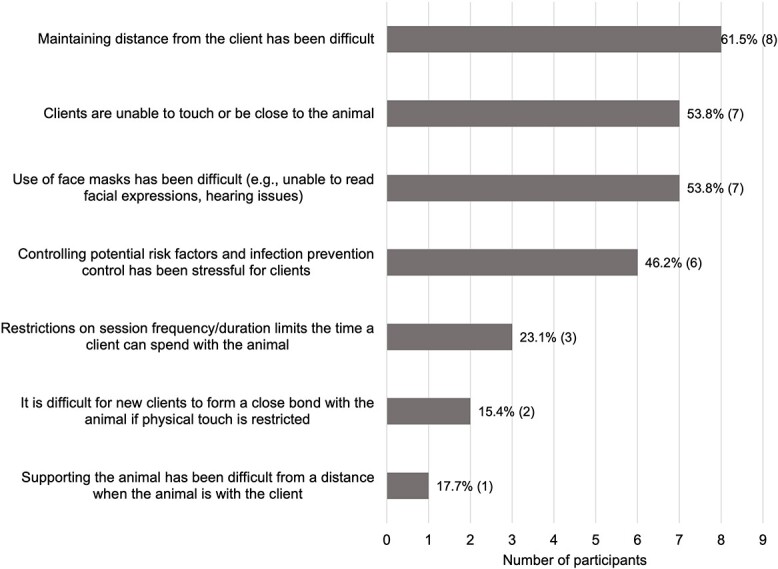
Providers perceptions of challenges when providing AAIs *in situ*.

Since the Covid-19 outbreak, the majority of providers (83.3%; *n* = 30) reported they were still delivering AAIs, though the frequency had decreased. Seventeen (56.7%) offered remote AAIs only and 11 (36.7%) offered a combination of remote and *in situ* delivery. Of these, all providers reported at least one challenge in relation to remote delivery (Fig. 1). Of those who continued to offer *in situ* interventions (43.3%; *n* = 13), all providers also reported at least one challenge (Fig. 2).

Of the thirty providers still delivering AAIs during the pandemic, twelve (40%) reported their animal was missing face-to-face interaction and four (13.3%) reported their animal’s behaviour had changed due to an altered work routine. However, 12 (40%) reported there had been no change in their animal. Sixteen providers (53.3%) reported they were less satisfied with the change in practice, followed by 12 (40%) indicating they felt the same. Only two providers indicated they were more satisfied.

### Perspectives regarding AAI provision during Covid-19

Twelve providers responded to the free-text question. Thematic analysis of responses resulted in the identification of two themes:

#### Advantages of transitioning to remote-delivery

Despite many challenges, there was a consensus that remote delivery had offered alternative ways to work towards client goals, worked well for those who were allergic/fearful of animals and increased the range of activities offered.

‘I have gained more ideas of activities as we have to think outside the box. The list of activities we now have is long and all sessions have been positively received. If and when we go back in person, these will be useful activities in our toolbox.’

Many providers suggested remote delivery was advantageous for future use for those who may be unable to attend sessions *in situ*. Some expressed that a hybrid approach may be beneficial *‘to target the exact needs and preferences of individuals’.*

#### Disadvantages associated with remote-delivery

Four providers also mentioned challenges. These included difficulties engaging with certain populations remotely, lack of staff available to support provision and client fatigue from excessive use of online activities. One provider highlighted the importance of sensory benefits when delivering AAIs and suggested the absence of physical touch during virtual interventions may be a disadvantage.

‘Clients have been unable to touch and play with the animals while the AAI was remotely operated. Some of the sensory aspect was lost, the physicality of the animal breathing while being held or crunching a vegetable is something that even the least able of clients can benefit from.’

## Discussion

### Main finding of the study

This survey has highlighted the impact of Covid-19 on AAI delivery, associated challenges and opportunities. Most of the providers who continued to deliver AAIs remotely during the pandemic identified restrictions of implementation, including clients being unable to touch the animal, and clients having restricted access to the required technology. However, advantages to remote delivery were also reported, such as reaching those who were allergic or fearful of animals. Commonly reported challenges of *in situ* delivery included difficulty maintaining distance from the client, the use of face masks perceived to hinder communication and the client being unable to touch or be close to the animal. It is possible that a hybrid approach may be beneficial to tailor the delivery to the needs and preferences of the individual. However, it is essential to consider animal welfare concerns alongside client needs when working remotely.^3^ For example, training is available to guide the animal to connect with the screen, but the handler must be cognizant of the effect it may have on the animal, especially over time.^18^

### What is already known on this topic

The positive effects of AAIs have been frequently reported in the literature for a range of populations, such as people with dementia,^19^ children^20^ and those with mental health conditions.^21^ Our findings support research reporting remote AAIs may still have a positive impact on clients,^12–15^ and may be particularly beneficial when the involvement of real animals is unrealistic or prohibited.^11^

Commonly cited hypothesized mechanisms of AAIs that may contribute to positive health outcomes include the animal’s personality, novel distraction and physical touch.^22^ Research reports that the physical interaction with an animal can provide tactile comfort, decrease tension and facilitate feelings of safety in the participant’s own environment.^10^^,^^22^^,^^23^ This aligns with our current findings, with providers indicating that the absence of physical touch was a perceived restriction in both modes of delivery, as clients receiving *in situ* interventions were still unable to be close to the animal within the pandemic context. However, providers also reported advantages to remote AAI delivery, suggesting physical touch may not be essential for positive outcomes to occur. Physical touch is only one of several potential active mechanisms during AAI delivery, and it may be a combination of mechanisms inherent to human–animal interaction that contributes to the overall benefits that result from these interventions.^22^ It remains crucial for future research to investigate theoretical frameworks and potential mechanisms underpinning AAIs to advance the field’s understanding of implementation and maximize the benefits in both remote and *in situ* delivery.

### What this study adds

The findings of this survey contribute to our understanding of AAI implementation and highlight the importance of conducting further research in this area and developing targeted strategies to lessen or overcome the associated challenges with delivering remote AAIs. The necessary transition to remote delivery has highlighted the potential for remote engagement with AAIs in the future, outside of a pandemic context. This may be particularly beneficial for certain populations, such as those living in remote communities or have allergies or fears of animals. Therefore, it is important for future research to further explore remote engagement and ensure the associated challenges can be overcome, particularly as society gains increased experience and familiarity with remote approaches. Future research should involve process evaluations, through which attention is paid to the barriers and facilitators of remote implementation. In addition, assessing the differences in outcomes on human health and animal welfare achieved by remote and *in situ* AAIs, respectively, would be an important research goal.

### Limitations

A limitation of this survey is the small sample of UK AAI providers included. This was perhaps surprising given our wide-reaching distribution methods. However, the provision of AAIs is not as common a practice in the UK compared with other global areas, which may have influenced the sample size. Due to the limited number of participants, the findings are not generalizable, but do contribute to a preliminary understanding of AAI provision in the given context and the potential for remote engagement with AAIs in the future. Secondly, given the process used by Qualtrics and the distribution methods used to recruit participants (e.g. via social media platforms), it was not possible to determine the number of survey invites that were distributed, or the number of potential participants who were aware of the survey and did/did not complete it (i.e. the response rate). Therefore, there may be a risk of under- or over-estimating the proportion of the providers that share the views reported in this small-scale study.

Lastly, as the purpose of the survey was to collect information about the perspectives of UK AAI providers, we did not obtain data from those receiving the interventions. Future research should explore the perspectives of those attending the interventions, as this would further validate the perceptions of the AAI providers. Furthermore, it would be beneficial to explore the differences between various client groups, as the findings are likely to differ across different populations (e.g. elderly participants, children, ethnic minority groups, those with mental health conditions). Nevertheless, these findings contribute to our understanding of remote AAIs and the associated challenges and opportunities, thus strengthening the evidence base for AAI implementation and future research design.

## Data Availability

The data underlying this article are available in OSF, at: https://osf.io/b35vj/.

## Notes

**Emily Shoesmith,** Research Fellow

**Selina Gibsone,** Research and Development Manager

**Elena Ratschen,** Reader (Associate Professor)
